# Heterosis in the freezing tolerance, and sugar and flavonoid contents of crosses between *Arabidopsis thaliana* accessions of widely varying freezing tolerance

**DOI:** 10.1111/j.1365-3040.2008.01800.x

**Published:** 2008-06

**Authors:** MARINA KORN, SILKE PETEREK, HANS-PETER MOCK, ARND G HEYER, DIRK K HINCHA

**Affiliations:** 1 Max-Planck-Institut für Molekulare Pflanzenphysiologie, Am Mühlenberg 1D-14476 Potsdam, Germany; 2 Leibniz-Institut für Pflanzengenetik und Kulturpflanzenforschung (IPK)Corrensstr. 3, D-06466 Gatersleben, Germany; 3 Biologisches Institut, Abt. Botanik, Universität StuttgartPfaffenwaldring 57, D-70569 Stuttgart, Germany

**Keywords:** cold acclimation, compatible solutes

## Abstract

Heterosis is defined as the increased vigour of hybrids in comparison to their parents. We investigated 24 F_1_ hybrid lines of *Arabidopsis thaliana* generated by reciprocally crossing either C24 or Col with six other parental accessions (Can, Co, Cvi, L*er*, Rsch, Te) that differ widely in their freezing tolerance. The crosses differed in the degree of heterosis for freezing tolerance, both in the non-acclimated state and after a 14 d cold acclimation period. Crosses with C24 showed more heterosis than crosses with Col, and heterosis was stronger in acclimated than in non-acclimated plants. Leaf content of soluble sugars and proline showed more deviation from mid-parent values in crosses involving C24 than in those involving Col, and deviations were larger in acclimated than in non-acclimated plants. There were significant correlations between the content of different sugars and leaf freezing tolerance, as well as between heterosis effects in freezing tolerance and sugar content. Flavonoid content and composition varied between accessions, and between non-acclimated and acclimated plants. In the crosses, large deviations from the mid-parent values in the contents of different flavonols occurred, and there were strikingly strong correlations between both flavonol content and freezing tolerance, and between heterosis effects in freezing tolerance and flavonol content.

## INTRODUCTION

Freezing tolerance is a primary factor that defines the geographic distribution of plants. In addition, it has a strong influence on the yield of crop plants in large parts of the world, where frost can lead to periodic catastrophic yield losses. Many plants from temperate and cold climates, including many important crop species, increase in freezing tolerance during exposure to low, but non-freezing temperatures in a process termed cold acclimation (see Thomashow 1999; Xin & Browse 2000; Smallwood & Bowles 2002 for comprehensive reviews). To understand the molecular basis of plant freezing tolerance, a strong emphasis has been on molecular analyses in the model plant *Arabidopsis thaliana*.

Plant freezing tolerance is a multigenic, quantitative trait, and gene expression profiling with whole genome arrays indicates that cold acclimation in *A. thaliana* involves changes in the expression levels of several hundred genes (Hannah, Heyer & Hincha 2005; Vogel *et al.* 2005; Hannah *et al.* 2006; Kaplan *et al.* 2007), while metabolite profiling revealed changes in the content of a large portion of cellular metabolites (Cook *et al.* 2004; Kaplan *et al.* 2004, 2007; Hannah *et al.* 2006). While many of these changes are probably associated with specific adaptations in metabolic pathways to low growth temperature (Guy *et al.* 2008), at least some metabolites may also function as compatible solutes.

Compatible solutes are synthesized by many organisms ranging from bacteria to animals and plants, in response to desiccation, osmotic stress, salt stress or low temperature. This chemically heterogeneous group of substances comprises some amino acids (e.g. proline), quaternary ammonium compounds (e.g. betaine), many sugars, sugar alcohols and several others (see Yancey *et al.* 1982; Somero 1992 for reviews). Physiologically, compatible solutes should have no adverse metabolic effects even at very high concentrations. They are thought to stabilize sensitive cellular components under stress conditions and also act as bulk osmoprotectants. Therefore, they may act colligatively by increasing the osmotic potential and thereby improving the water status, and therefore, the cell volume in the frozen state. In addition, they can stabilize macromolecular structures such as proteins by preferential exclusion from the hydration shell of proteins (Timasheff 1993), assist refolding of unfolded polypeptides by chaperone proteins (Diamant *et al.* 2001) and stabilize membranes during freezing and drying (Crowe *et al.* 1990; Hincha, Popova & Cacela 2006). However, the contribution of different compatible solutes such as sugars and proline to the freezing tolerance and cold acclimation of *Arabidopsis* and other plant species has not yet been resolved (see Hincha *et al.* 2005 for a recent review).

Attempts to understand the genetic and molecular basis of complex quantitative traits in plants have in recent years focused on the analysis of natural genetic variation. *Arabidopsis thaliana* is a geographically widely spread species, and it has been shown that different accessions have sufficient genetic variability to allow investigations of genotype × environment interactions (see Koornneef, Alonso-Blanco & Vreugdenhil 2004; Mitchell-Olds & Schmitt 2006 for reviews). This is also true for freezing tolerance, where a clear correlation with both latitude of origin and habitat growth temperature has been shown (Hannah *et al.* 2006; Zhen & Ungerer 2008). Recently, quantitative trait locus (QTL) mapping was successfully employed to gain insight into the molecular basis of the differences in acclimated freezing tolerance between the accessions Cape Verde Islands (Cvi) and Landsberg *erecta* (L*er*) (Alonso-Blanco *et al.* 2005). In addition, crosses of *Arabidopsis* accessions can be used to create further genetic and phenotypic variation through heterosis effects (see e.g. Griffing & Scholl 1991; Narang & Altmann 2001; Barth *et al.* 2003; Meyer *et al.* 2004).

The analysis of such heterotic offspring could lead to new insights into the molecular basis of plant freezing tolerance. In addition, in an almost exclusively selfing species like *A. thaliana* (Abbott & Gomes 1989), accessions are largely homozygous, and this is expected to lead to inbreeding depression. Crossing such accessions leads to increased heterozygosity, which may result in heterosis. The term heterosis (Shull 1914) describes the phenomenon of increased physiological performance of F_1_ hybrids in comparison to their parents. This can be expressed either as mid-parent heterosis (MPH), or best-parent heterosis (BPH). MPH denotes the deviation of the F_1_ from the parental mean and can be either positive or negative. BPH denotes cases where the F_1_ outperforms the better parent and can, by definition, only be positive. Although heterosis has been used extensively by breeders to increase the performance of crop plants, our understanding of its molecular basis is still rudimentary (see Birchler, Auger & Riddle 2003; Hochholdinger & Hoecker 2007 for reviews).

In a recent publication, we have shown that both MPH and BPH can be observed in the freezing tolerance of reciprocal crosses of the *Arabidopsis* accessions C24 and Col (Rohde, Hincha & Heyer 2004). Here, we demonstrate that heterosis in *Arabidopsis* freezing tolerance is not restricted to this combination of genotypes, but that it occurred in a wide range of crosses involving C24, while the heterosis effects were generally smaller in crosses involving Col. There were also large deviations from mid-parent values in the content of several sugars and in the content of different flavonols. The content of some, but not all, of these compounds correlated strongly with freezing tolerance in the accessions and crosses. In addition, we showed for the first time strong correlations between the heterosis effects in freezing tolerance and the content of some of these compounds, suggesting possible causative relationships.

## MATERIALS AND METHODS

### Plant material

We used *A. thaliana* plants from the accessions C24, Canary Islands (Can), Coimbra-2 (Co), Columbia-0 (Col), Cape Verde Islands (Cvi), Landsberg *erecta* (L*er*), Rschew (Rsch) and Tenela (Te). The sources of the different seed stocks have been described in a recent publication (Schmid *et al.* 2006). Seeds for our experiments have been generated through single seed descent to assure genetic homogeneity of the plants (Törjek *et al.* 2003). Plants were grown in soil in a greenhouse at 16 h day length with light supplementation to reach at least 200 *µ* E m^–2^ s^–1^ and a temperature of 20 °C during the day, 18 °C during the night until bolting (compare Hannah *et al.* 2006). For cold acclimation, plants were transferred to a 4 °C growth cabinet at 16 h day length with 90 *µ* E m^–2^ s^–1^ for an additional 14 d. The reduced light intensity avoids the risk of photoinhibitory damage at the low temperature, but is sufficient to allow cold acclimation, including the accumulation of sugars and starch (Rohde *et al.* 2004; Zuther *et al.* 2004; Hannah *et al.* 2006; Guy *et al.* 2008).

### Freezing experiments

Freezing damage was determined as electrolyte leakage after freezing of detached leaves to different temperatures as described in detail in previous publications (Rohde *et al.* 2004; Hannah *et al.* 2006). Between 12 and 24 plants were analysed in each experiment from each genotype and treatment. All experiments were performed at least twice. The LT_50_ was calculated as the LOGEC50 value of unanchored sigmoidal curves fitted to the leakage values using the software GraphPad Prism3. Statistical analysis of the differences in LT_50_ values was performed using *t*-tests with GraphPad Instat software. Analyses of variance (anovas) were performed with the statistics package in the KaleidaGraph software (Synergy Software, Reading, PA, USA).

### Carbohydrate analysis

Three leaves from individual plants that were also used in the freezing experiments were harvested at midday (6–8 h after lights on). They were frozen in liquid nitrogen immediately after sampling and homogenized using a ball mill Retsch MM 200 (Retsch, Haan, Germany). Soluble sugars were extracted and analysed by high-performance liquid chromatography (HPLC) as described previously (Rohde *et al.* 2004). Statistical analysis was performed using *t*-tests in Excel.

### Proline measurements

Proline measurements were performed photometrically as previously described (Bates, Waldren & Teare 1973; Rohde *et al.* 2004) on the same leaf samples that were also used for sugar measurements. Statistical analysis was performed using *t*-tests in Excel.

### Analysis of flavonoids by liquid chromatography–mass spectrometry (LC–MS)

Powdered plant material was extracted twice with 80% methanol at a ratio of 1:8 (leaf fresh weight : volume) and cleared by centrifugation. Phenylpropanoids were separated by reversed-phase HPLC (Acquity UPLC with a BEH C18, 1.7 *µ* m, 2.1 × 50 mm column; Waters, Eschborn, Germany) at 25 °C. The mobile phase was composed of 98% water, 2% formic solution (= 5% ammonium formate in formic acid) (solvent A) and 100% acetonitrile (solvent B). Flavonoids were eluted with the following gradient at a flow rate of 0.6 mL min^–1^: initial, 0% B; 1.0 min, 0% B; 3.5 min, 40% B; 4.0 min, 100% B; 4.25 min, 100% B; 4.5 min, 0% B; 5.0 min, 0% B.

Eluted substances were detected with a photodiode array detector (PDA 2996; Waters). Ten absorbance spectra were recorded every second, between 210 and 600 nm, with a bandwidth of 1.2 nm, and chromatograms were extracted from the PDA data at 280 nm. Data were analysed using Waters MassLynx software.

The eluted substances were identified using an electrospray ionization–time-of-flight (ESI–ToF) mass spectrometer (LCT Premier; Waters). The mass spectrometer operated in a positive ion mode with a source temperature of 100 °C and a cone gas flow of 5.0 L h^–1^. The mass spectra were acquired with the mass analyser in W-mode, and spectra were integrated over 1 s intervals. MS data were acquired in a continuum mode using MassLynx 4.1 software (Waters). The instrument was calibrated with a multi-point calibration using selected fragment ions of phosphoric acid. Leucine enkephalin was used as lockspray reference probe.

## RESULTS

### Heterosis in the freezing tolerance of crosses between *Arabidopsis* accessions

**Figure 1:**
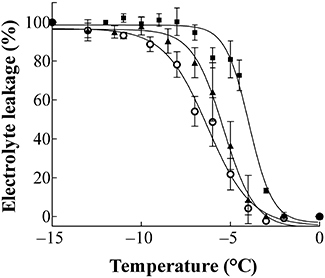
Electrolyte leakage from *Arabidopsis thaliana* leaves after freezing and thawing. Leaves from non-acclimated plants of the accessions C24 (solid squares) and Co (solid triangles), and a cross between the two accessions (C24 × Co; open circles) were frozen to different temperatures. The electrolyte leakage values are the means ± standard error of the mean (SEM) from three measurements. Sigmoidal curves were fitted to the data to determine the LT_50_ values (temperature of 50% electrolyte leakage), which were –4.0 ± 0.15 °C for C24, –5.4 ± 0.22 °C for Co and –6.3 ± 0.22 °C for the C24 × Co F_1_ plants.

We determined the freezing tolerance of *Arabidopsis* by slowly freezing detached leaves to different temperatures. After thawing, electrolyte leakage was determined with a conductimeter, and from the fitted sigmoidal curves the LT_50_ (temperature of 50% electrolyte leakage) was calculated as a quantitative measure of leaf freezing tolerance (Rohde *et al.* 2004; Hannah *et al.* 2006). Figure 1 shows as an example the electrolyte leakage curves for non-acclimated plants (NA) of the accessions C24 and Co, and of one of the reciprocal crosses (C24 × Co). As noted before (Hannah *et al.* 2006), Co was more freezing tolerant than C24 (LT_50_ = –5.4 and –4.0 °C, respectively). The F_1_ cross of these two parental accessions was more freezing tolerant than either parent (LT_50_ = –6.3 °C), resulting in significant MPH (27%) and BPH (23%), similar to the situation previously reported for C24 and Col (Rohde *et al.* 2004).

**Figure 2:**
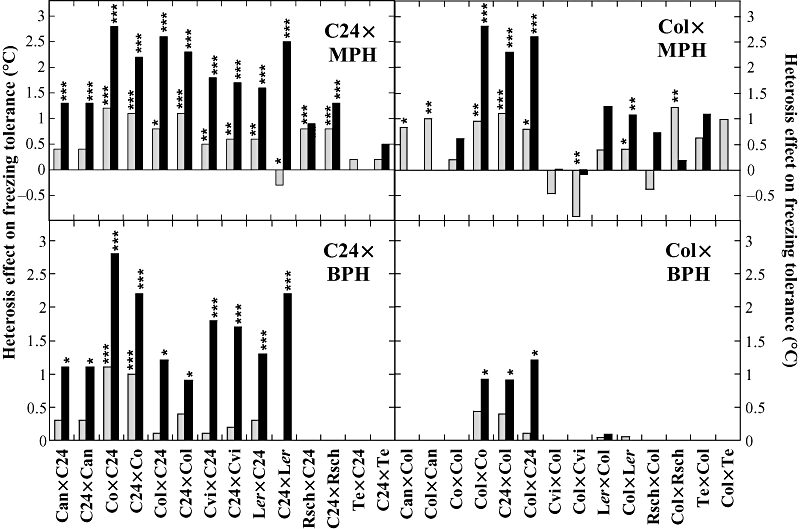
Heterosis effects on the freezing tolerance of leaves from F_1_ plants generated by systematically crossing the accessions C24 and Col with a panel of accessions varying in freezing tolerance. Leaves were harvested either from non-acclimated (grey bars) or cold-acclimated (black bars) plants. Heterosis was calculated either as mid-parent heterosis (MPH) or as best-parent heterosis (BPH). Analysis of variance (anova) analysis showed a significant influence of genotype on heterosis (*F* = 5.04; *P* < 0.0001), and a significant effect of cold acclimation on both MPH (*F* = 19.77; *P* < 0.0001) and BPH (*F* = 12.10; *P* = 0.0011). The significance of the heterosis effects in the different F_1_ populations was determined by *t*-test and is indicated by the asterisks above the bars (*P ≤ 0.05; **P ≤ 0.01; ***P ≤ 0.001). The data for the C24 × Col and Col × C24 plants were taken from Rohde *et al.* (2004).

It is generally not clear which parental properties, such as variation in parental phenotypical or genetical differences, determine the magnitude of heterotic effects. Therefore, we performed systematic manual pollination experiments to cross the already characterized accessions C24 and Col with a panel of six accessions varying widely in freezing tolerance (non-acclimated LT_50_ between –4.5 °C for C24 and –7.1 °C for Te; acclimated LT_50_ between –6.2 °C for Co and Can, and –12.1 °C for Te; Hannah *et al.* 2006) and geographic origin (16° to 66° northern latitude; Hannah *et al.* 2006). For all reciprocal crosses and their parents, freezing experiments were performed as described in Fig. 1, both before and after cold acclimation for 14 d at 4 °C. Figure 2 shows the heterosis effect on freezing tolerance for all 28 crosses, including the previously published data (Rohde *et al.* 2004) for C24 × Col and Col × C24 for comparison. Statistical analysis showed a highly significant influence of genotype on the magnitude of the heterosis effect (*F* = 4.59; *P* < 0.0001; Fig. 2), validating this approach.

Two conclusions are immediately apparent from these data. Heterosis was always larger in acclimated than in non-acclimated plants, and in general heterosis effects were bigger and more frequent in crosses involving C24 than in crosses involving Col. The highest relative MPH and BPH were found in Co × C24 with 27 and 23% for non-acclimated plants, and with 44% for both MPH and BPH for acclimated plants. Crosses involving Col reached a maximum of 18% MPH in the non-acclimated state (Col × Co) and 31% (Col × Co) in the acclimated state. Significant BPH was only observed after cold acclimation and reached a maximum of 23% in Col × C24.

While in most combinations of accessions the two reciprocal crosses showed very similar levels of heterosis, there were some remarkable exceptions. The two most striking ones are the combinations of C24 and L*er*, and of Col and Co (Fig. 2). Interestingly, these presumed maternal effects could also depend on the paternal genotype, as the reciprocal combinations of Col and L*er* showed no corresponding differences.

**Figure 3:**
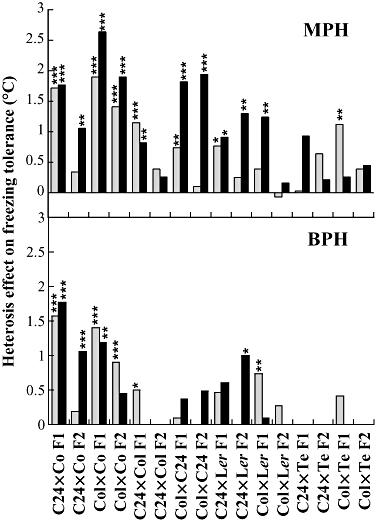
Heterosis effects on the freezing tolerance of leaves from F_1_ plants generated by crossing the accessions C24 and Col with a subset of the accessions shown in Fig. 2 and the respective F_2_ plants generated by selfing the F_1_ plants. Leaves were harvested either from non-acclimated (grey bars) or cold-acclimated (black bars) plants. Heterosis was calculated either as mid-parent heterosis (MPH) or as best-parent heterosis (BPH). The significance of the heterosis effects was determined by *t*-test and is indicated by the asterisks above the bars (compare Fig. 2).

Because heterotic effects are by definition (Shull 1914) based on heterozygosity in the F_1_ plants, further inbreeding should reduce heterosis. For a selected panel of crosses, we have therefore obtained F_2_ plants by selfing the respective F_1_ plants and compared the heterotic effects on freezing tolerance (Fig. 3). In most cases, a reduction in heterosis was apparent. This indicates that the F_2_ populations showed segregation towards transgression to less freezing-tolerant phenotypes. However, in C24 × L*er*, heterosis of acclimated plants increased in the F_2_ compared to the F_1_. This is not related to the maternal effect observed in this cross, because the C24 × Co cross showed the expected reduction of heterosis in the F_2_ (Fig. 3). It is therefore possible that the heterosis in the C24 × L*er* cross is caused by the stable complementation of a defective gene(s) rather than increased heterozygosity.

### Compatible solutes in the different genotypes

During plant cold acclimation, the content of most solutes in leaf cells increases (Cook *et al.* 2004; Kaplan *et al.* 2004, 2007; Hannah *et al.* 2006), and there is evidence from several studies that at least some of these solutes may be important for the development of freezing tolerance (see Xin & Browse 2000; Smallwood & Bowles 2002 for reviews). We have therefore measured the amounts of four sugars (Fru, Glc, Suc, Raf) and the amino acid proline in leaf samples from all parental accessions and their reciprocal F_1_ hybrids, both before and after cold acclimation.

**Figure 4:**
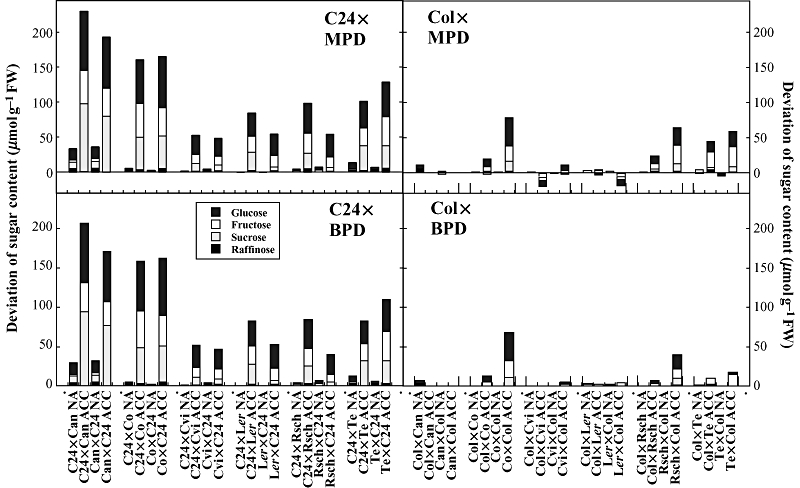
Deviations from mid-parent or best-parent values in the content of soluble carbohydrates in the leaves of the F_1_ plants characterized for freezing tolerance in Fig. 2. Leaves were harvested either before (NA) or after (ACC) cold acclimation. Analysis of variance (anova) showed a significant influence of genotype on heterosis for all sugars (Glc: *F* = 77.11; Fru: *F* = 36.99; Suc: *F* = 618.43; Raf: *F* = 16.74; *P* < 0.0001 for all sugars). The statistical significance of all deviations in sugar content is shown in Supplementary Table S1.

The data were used to calculate deviations from mid-parent and best-parent values (i.e. heterosis effects) in the sugar content of all F_1_ plants investigated in this study (i.e. not for the crosses between C24 and Col, where this has been published previously; Rohde *et al.* (2004). Because the term heterosis is used in the context of traits like seed yield, biomass or stress tolerance (Hochholdinger & Hoecker 2007), we used the terms mid-parent deviation (MPD) and best-parent deviation (BPD) in the remainder of this paper for the content of different substances in the plants. They were calculated identically to MPH and BPH in freezing tolerance, and therefore allow quantitative comparisons. We found deviations from both mid-parent and best-parent values for the content of all four sugars (Fig. 4) in a pattern similar to that obtained for the heterosis in freezing tolerance (Fig. 2). These deviations in sugar content depended significantly on genotype and were much larger and more frequent in cold-acclimated than in non-acclimated plants. Furthermore, the effects were stronger and more frequent in crosses involving C24 than in those involving Col. Interestingly, the differences in heterosis effects between some reciprocal crosses observed for freezing tolerance described earlier were not reflected in the corresponding sugar contents. Supplementary Table S1 gives an overview of the statistical significance of all MPD and BPD values.

**Figure 5:**
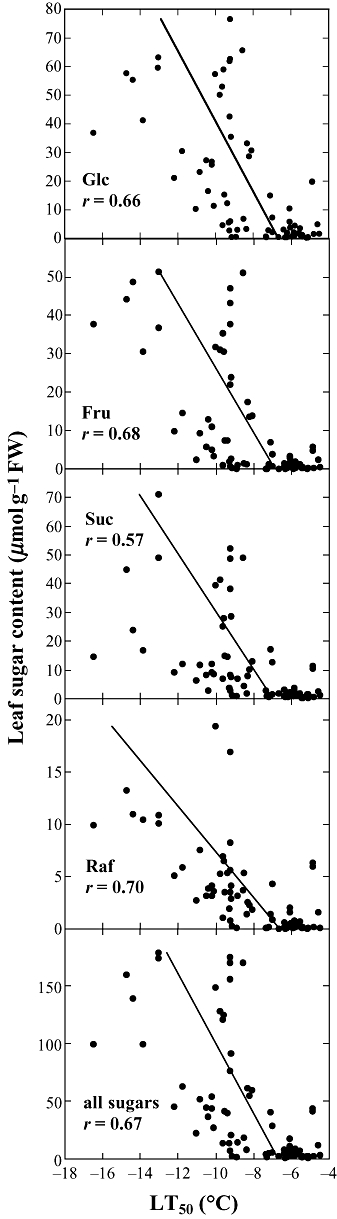
Analysis of the correlations between the content of different soluble sugars in the leaves and leaf freezing tolerance. Data were compiled from all investigated accessions and their F_1_ for both non-acclimated and cold-acclimated plants. For the bottom panel (all sugars), the content of Glc, Fru, Suc and Raf was added up for each sample. The lines were fitted to the data by least square linear regression analysis, and the correlation coefficients are shown in the panels. The *P* values for all correlations were below 0.0001.

To obtain insight into the functional significance of the accumulated sugars in freezing tolerance as such and in heterosis for freezing tolerance, we first correlated sugar content with LT_50_ (Fig. 5). The content of all four sugars showed a relatively good correlation with LT_50_ (from *r* = 0.57 for sucrose to *r* = 0.70 for raffinose; all *P* values below 0.0001). The correlation was of similar quality (*r* = 0.67) when the content of all sugars was added up (bottom panel in Fig. 5), indicating that the individual sugars may not play highly specific roles in leaf freezing tolerance.

**Figure 6:**
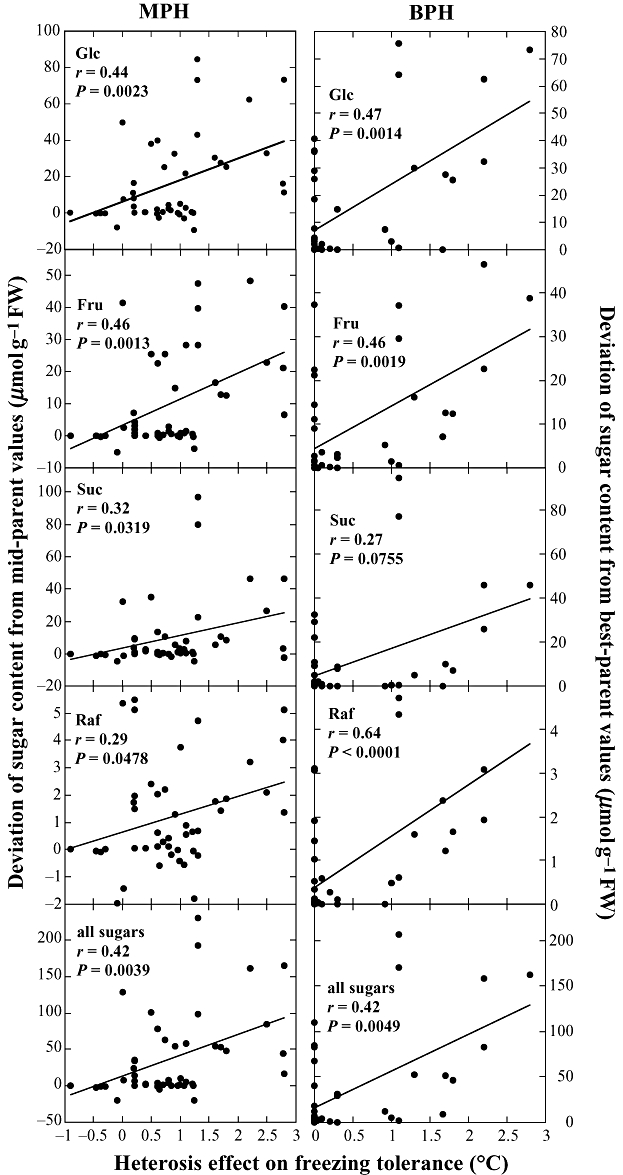
Analysis of the correlations between deviations from mid-parent or best-parent values in the content of different soluble sugars in the leaves and the heterosis effects on leaf freezing tolerance (compare Figs 2 & 4). Data were compiled from all investigated accessions and their F_1_ for both non-acclimated and cold-acclimated plants, and include both statistically significant and non-significant effects. The lines were fitted to the data by least square linear regression analysis, and the correlation coefficients and *P* values are shown in the panels. Analyses were performed for both mid-parent heterosis (MPH, left panels) and best-parent heterosis (BPH, right panels) in freezing tolerance.

Similarly, the role of differences in sugar content in the establishment of heterosis in freezing tolerance was evaluated by correlating heterosis effects in LT_50_ with the respective MPD and BPD for sugar content, separately for MPH and BPH (Fig. 6). In general, these correlations were weaker than those between LT_50_ and sugar content, as indicated by both the *r* and *P* values (compare Fig. 5), and they were better for BPH than for MPH. MPD and BPD in sucrose content showed the lowest correlations with heterosis in freezing tolerance, and MPD in raffinose content also showed a surprisingly low correlation. This correlation, however, was much better when BPD was considered. For both MPD and BPD, the correlations obtained after adding up the effects of all sugars were within the range of the correlation coefficients for the separate effects of the individual sugars, again suggesting redundancy between the sugars.

**Figure 7:**
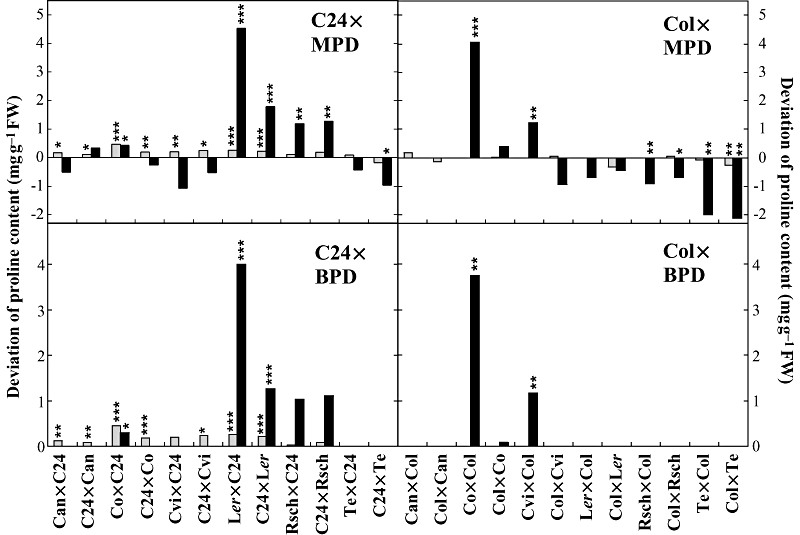
Deviation from mid-parent (MPD) and best-parent (BPD) values of proline content in the leaves of the F_1_ plants characterized for freezing tolerance in Fig. 2. Leaves were harvested either before (grey bars; NA) or after (black bars; ACC) cold acclimation. Analysis of variance (anova) showed a significant influence of genotype on heterosis (*F* = 2.90; *P* = 0.00077). Significance of the heterosis effect in the different F_1_ populations was determined by *t*-test and is indicated by the asterisks above the bars (compare Fig. 2).

**Figure 8:**
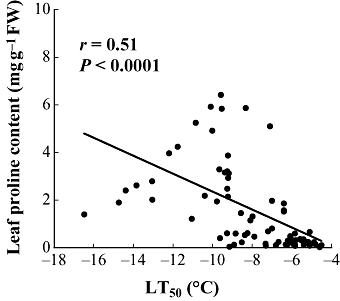
Analysis of the correlation between the proline content of *Arabidopsis* leaves and leaf freezing tolerance. Data were compiled from all investigated accessions and their F_1_ for both non-acclimated and cold-acclimated plants. The line was fitted to the data by least square linear regression analysis, and the correlation coefficient and *P* value are shown in the panel.

Another potentially beneficial solute that *Arabidopsis* accumulates during cold acclimation is proline. Figure 7 shows that deviations from mid-parent or best-parent values were less frequent for proline than for sugar content (compare Fig. 4), but still depended significantly on genotype. Several of the F_1_ plants derived from crosses with Col showed strong negative MPD. Consequently, while there was a moderate, but significant correlation between LT_50_ and proline content of the investigated genotypes (Fig. 8), there was no significant correlation between either MPD or BPD in proline content, and MPH or BPH in freezing tolerance (data not shown), indicating that differential proline accumulation played no major role in the establishment of heterosis effects in *Arabidopsis* freezing tolerance.

### Flavonoid content and composition in the different genotypes

One of the major biochemical pathways in *Arabidopsis* that is strongly cold induced at the transcript level is flavonoid biosynthesis (Hannah *et al.* 2005, 2006). We used LC–MS technology to comprehensively profile the flavonoid composition of a subset of five accessions and eight F_1_ crosses, both before and after cold acclimation. This subset of accessions and crosses covers the whole range of LT_50_ values and heterosis effects in LT_50_ determined in the larger selection described earlier.

**Figure 9:**
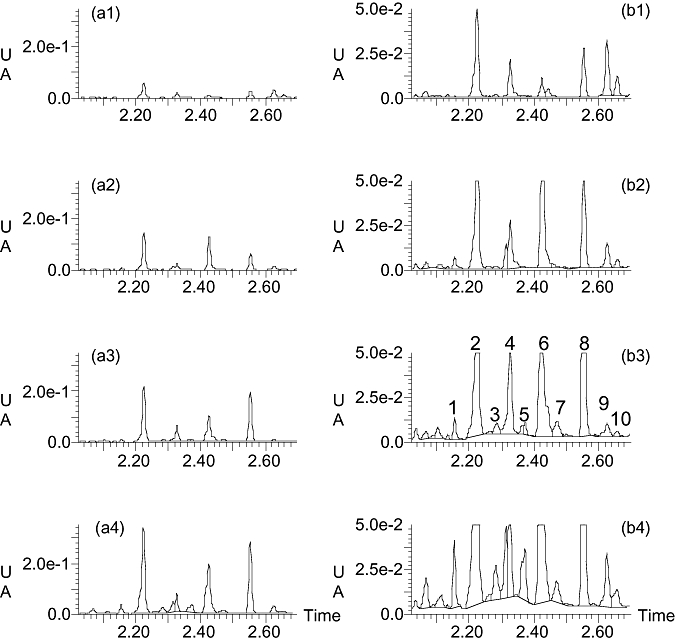
High-performance liquid chromatography (HPLC) analysis of flavonoids from leaves of the *Arabidopsis* accessions C24 (a1/a2 and b1/b2) and Te (a3/a4 and b3/b4) before (a1/b1 and a3/b3) and after (a2/b2 and a4/b4) cold acclimation. Absorbance spectra were taken with a diode array detector, and absorbance at 280 nm is plotted in the figure. In addition, the peaks numbered 1–10 in panel b3 were analysed by mass spectroscopy, and both absorbance and mass spectra were used to determine the substances underlying the peaks (see Table 1). The areas of peaks 2, 4, 6 and 8 were quantified to compare flavonoid content and composition (Figs 10–12). Right panels (b1–b4) are enlargements of the chromatograms shown in the corresponding left panels (a1–a4), revealing changes also for minor components.

Figure 9 shows the HPLC elution profiles of flavonoids extracted from the leaves of the accessions C24 and Te, which constitute the extremes in freezing tolerance in our selection of accessions (compare Hannah *et al.* 2006). Comparison of the four upper panels (a1/b1; a2/b2) with the four lower panels (a3/b3; a4/b4) reveals that the more freezing-tolerant accession Te contained higher amounts of flavonoids than the less freezing-tolerant accession C24. However, both accessions showed a clear increase in flavonoid content with cold acclimation (compare e.g. a1 with a2, and a3 with a4). An integration of the peak areas yielded quantitative data for further analysis.

**Table 1:** Identification of the flavonoids present in the peaks shown in the high-performance liquid chromatography (HPLC) elution profiles in Fig. 9

			Compound masses (M + H)^+^		
				
Peak number	Compound class	UV/Vis *λ*_max_ (nm)	ESI–MS (*m*/*z*)	MS fragments (*m*/*z*)	Predicted compounds
1	Flavonol derivative	256/356	757	454	Quercetin–Rha–Rha–Glc
2	Flavonol derivative	266/348	741	496	Not identified
3	Anthocyanin	527	Not determined (only detectable in Te)		Not identified
4	Flavonol derivative	266/347	757	611	Quercetin–Rha–Rha–Glc
5	Flavonol derivative	266/345	757	595/432	Kaempferol–Glc–Glc–Rha
6	Flavonol derivative	266/347	595	476	Kaempferol–Rha–Glc
7	Anthocyanin	544	Not determined (only detectable in Te)		
8	Flavonol derivative	265/345	579	433	Kaempferol–Rha–Rha
9	Cinnamic acid derivative	331	363		Sinapoyl malate
10	Cinnamic acid derivative	317			Not identified

Compounds were identified by photodiode array detector (PDA) absorbance spectra and mass spectrometry.

Rha, rhamnose; Glc, glucose.

**Figure 10:**
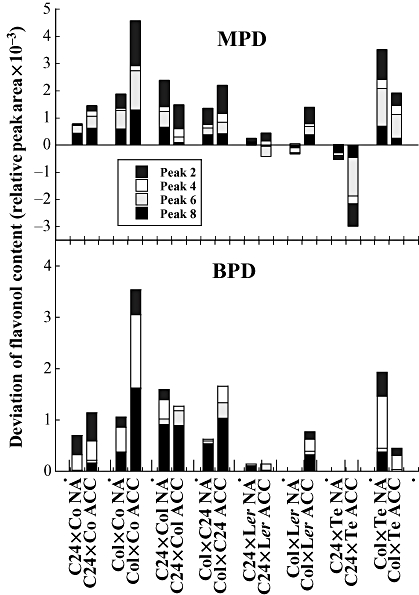
Deviation from mid-parent (MPD) and best-parent (BPD) values of the content of different flavonol glycosides in the leaves of selected *Arabidopsis* crosses. Leaves were harvested either before (NA) or after (ACC) cold acclimation. The peak numbering refers to the high-performance liquid chromatography (HPLC) elution profile shown in Fig. 9, b3. Further information about the analysed compounds can be found in Table 1. Analysis of variance (anova) showed a significant influence of genotype on heterosis for all flavonols (peak 2: *F* = 10.21, *P* < 0.0001; peak 4: *F* = 3.74, *P* = 0.014; peak 6: *F* = 5.55, *P* = 0.00098; peak 8: *F* = 3.83, *P* = 0.0067). The statistical significance of all deviations in flavonol content is shown in Supplementary Table S2.

The substances detected at 280 nm were characterized by absorbance spectroscopy and mass spectrometry. Ten peaks could be clearly resolved by HPLC (Fig. 9, b3). Peaks 3 and 7 were not further characterized, as they contained anthocyanins that could only be detected in extracts from cold-acclimated leaves from Te. Absorbance spectroscopy indicated that the minor peaks 9 and 10 contained cinnamic acid derivatives that were also not further characterized. Peaks 1, 2, 4, 5, 6 and 8 contained flavonol derivatives. Peaks 1 and 2 contained quercetin as the aglycon with different numbers of Glc and Rha molecules attached, while peaks 5, 6 and 8 contained kaempferol as the aglycon, and also Glc and Rha as sugar substituents (Table 1). For the quantitative analysis of leaf flavonoids, we only used the major peaks 2, 4, 6 and 8.

Several different F_1_ plants showed significant deviations from mid-parent or best-parent values in their content of various flavonols (Fig. 10), and the magnitude of both MPD and BPD for all flavonols depended significantly on plant genotype. Only the C24 × Te cross displayed strongly negative MPD, while the Col × Te cross showed positive MPD and also BPD. The crosses of C24 and Col with L*er* yielded no significant deviations for any flavonols (Supplementary Table S2), while the reciprocal crosses between C24 and Col, as well as the C24 and Col crosses with Co showed MPD and BPD.

**Figure 11:**
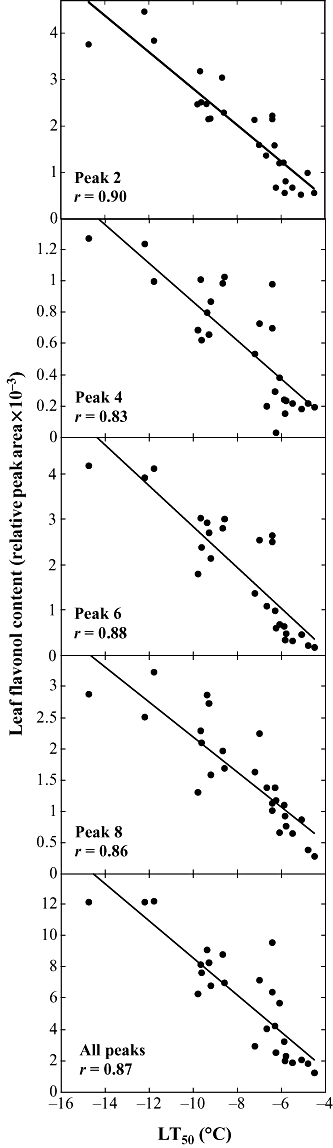
Analysis of the correlations between the flavonol content of *Arabidopsis* leaves and leaf freezing tolerance. Data were compiled from all investigated accessions and their F_1_ for both non-acclimated and cold-acclimated plants. For the bottom panel (all flavonols), the content of all four flavonol peaks (peaks 2, 4, 6 and 8; compare Fig. 9 and Table 1) was added up for each sample. The lines were fitted to the data by least square linear regression analysis, and the correlation coefficients are shown in the panels. The *P* values for all correlations were below 0.0001.

**Figure 12:**
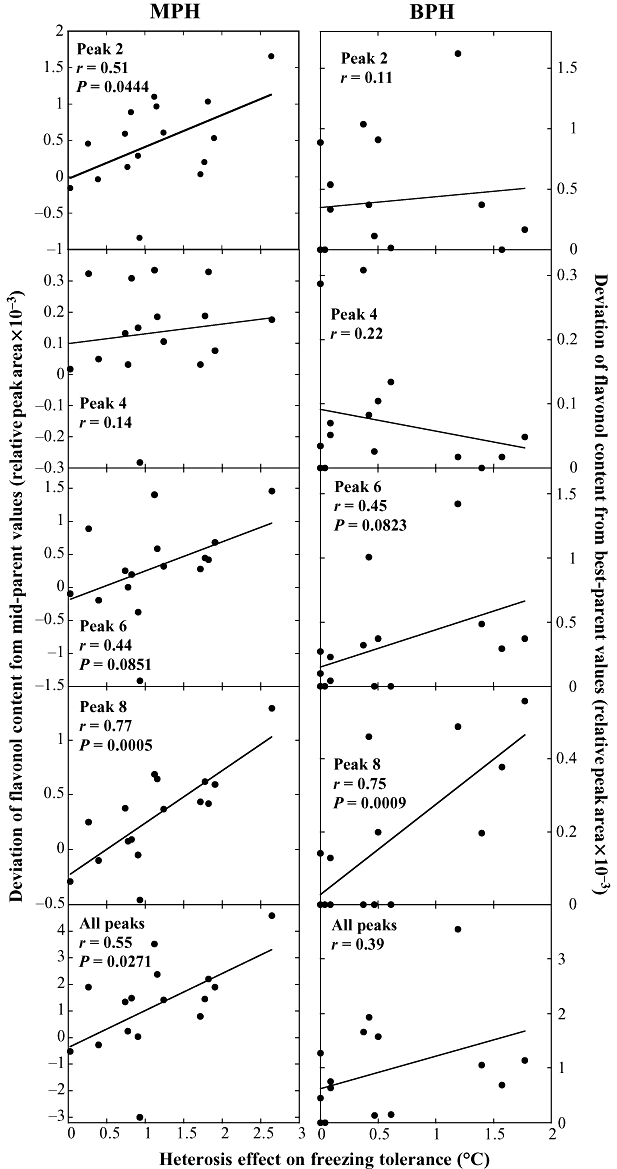
Analysis of the correlations between deviations from mid-parent or best-parent values in the content of different flavonols in the leaves and the heterosis effects on leaf freezing tolerance (compare Figs 2 & 10). Data were compiled from all investigated accessions and their F_1_ for both non-acclimated and cold-acclimated plants, and include both statistically significant and non-significant deviations. The lines were fitted to the data by least square linear regression analysis, and the correlation coefficients are shown in the panels. Correlation *P* values are only shown when they were smaller than 0.1. Analyses were performed for both mid-parent heterosis (MPH, left panels) and best-parent heterosis (BPH, right panels) in freezing tolerance.

The amounts of flavonols in the major peaks and the sum of all flavonols in these peaks were strongly (*r* values between 0.83 and 0.90; all *P* values below 0.0001) correlated with leaf freezing tolerance (Fig. 11). The correlations between heterosis in leaf freezing tolerance and deviations in flavonol content from mid-parent and best-parent values indicated specific roles for particular flavonols (Fig. 12). MPD and BPD in the content of quercetin glycosides (peak 4) showed no significant correlation with heterosis in freezing tolerance. The same was true for BPD in the content of the flavonol derivatives contained in peak 2, but not for MPD. The highest correlations, however, were found for the MPD and BPD of the kaempferol glycosides in peak 8 (*r* = 0.77 and 0.75, respectively). These correlations were much better than those obtained when the MPD or BPD of all flavonols was added up (*r* = 0.55 and 0.39), or those for peak 6, which contained a different kaempferol glycoside (*r* = 0.44 and 0.45). These differences in the *r* values were also reflected in the respective *P* values. This may indicate a more specific role of the flavonol (kaempferol–Rha–Rha) represented in peak 8.

## DISCUSSION

While heterosis in *Arabidopsis* freezing tolerance has been reported previously (Rohde *et al.* 2004) for crosses between C24 and Col, the present study extends this to 24 crosses representing reciprocal pairs of 12 combinations of accessions. The data show that C24 has a better combining ability than Col, as heterosis was much more frequent in crosses involving C24 than in those involving Col. Significantly, two of the three hybrids that showed BPH in crosses with Col were Col × C24 and C24 × Col described by Rohde *et al.* (2004). It is also interesting to note that heterosis in biomass production and heterosis in freezing tolerance were not genetically related. There are five crosses that have been investigated both for freezing tolerance in our study and for biomass production (Meyer *et al.* 2004), and the magnitudes of heterosis shown by these crosses differed strongly for the two traits. Clearly, it is not general ‘vigour’ that determines heterosis for these two traits, but very specific combinations of alleles in the F_1_ hybrids. The fact that in almost all cases heterosis decreased in the F_2_ plants compared to the F_1_ is in agreement with this hypothesis.

**Figure 13:**
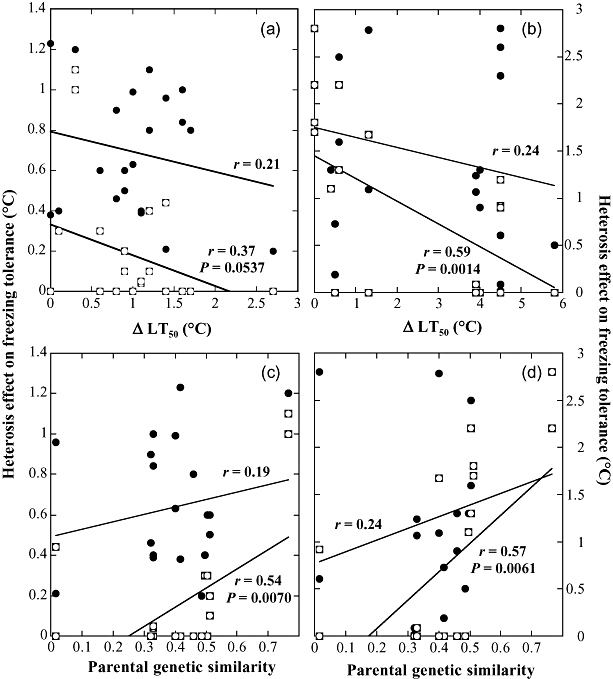
Dependence of the heterosis effect in leaf freezing tolerance on phenotypical (ΔLT_50_; difference in freezing tolerance between the parental accessions) and genotypical (parental genetic similarity) distance. Panels (a) and (b) show the data for phenotypical distances, and panels (c) and (d) the data for genotypical differences for non-acclimated (a, c) and cold-acclimated (b, d) plants. The solid symbols represent mid-parent heterosis (MPH) and the open symbols best-parent heterosis (BPH). All lines were fitted to the data by least square linear regression analysis, and the correlation coefficients are shown next to the lines. Correlation *P* values are only shown when they were smaller than 0.1.

It would be of considerable practical and theoretical interest to be able to predict the magnitude of the heterosis effect from characteristics of the parental plants. Such characteristics may be, for example, the phenotypic or genotypic similarity between parental lines. For phenotypic similarity, we found that the difference in freezing tolerance between the parental accessions and the magnitude of the heterosis effect in freezing tolerance of the respective F_1_ plants are in all four cases (MPH/NA, MPH/ACC, BPH/NA, BPH/ACC) negatively correlated (Fig. 13a,b). This is particularly pronounced for BPH in cold-acclimated plants (*r* = 0.59; *P* = 0.0014). It should also be mentioned at this point that the freezing tolerance of the F_1_ plants never exceeded that of the most freezing-tolerant parent in our study, Te. In fact, all crosses involving the two most freezing-tolerant accessions (Rsch and Te; Hannah *et al.* 2006) showed either no or very low heterosis. This could be because of the fact that these two accessions have particularly poor combining ability. However, crosses of C24 with Rsch and Te showed significant heterosis for biomass production (Meyer *et al.* 2004), making this explanation unlikely. Alternatively, there may be genetical constraints limiting freezing tolerance in *Arabidopsis*, that cannot be transgressed by crossing different accessions. The fact that the F_2_ populations showed reduced average heterosis compared to the F_1_ also argues against substantial transgression towards increased freezing tolerance.

To estimate parental genetic similarity, we used a distance matrix based on a pairwise comparison of genotypic data from 115 single nucleotide polymorphism (SNP)-based markers (Schmid *et al.* 2006). An analysis of the correlation between parental genetic similarity and the magnitude of the heterosis effects showed that in general heterosis was bigger in the genetically more similar combinations. The correlations were particularly strong for BPH in both acclimated and non-acclimated plants. This is again different from the situation for heterosis for biomass production, where a weak inverse correlation was found (Meyer *et al.* 2004), emphasizing again the basic genetical difference between heterosis in biomass production and freezing tolerance.

Metabolite profiling using GC–MS showed that the majority of detected metabolites was increased in content during cold acclimation (see Guy *et al.* 2008 for review). However, analysis of the freezing tolerance and metabolite content of nine different *Arabidopsis* accessions revealed a number of metabolites, particularly sugars, that were significantly correlated with acclimated freezing tolerance (Hannah *et al.* 2006). Here, we have analysed the sugar and proline content of 32 different genotypes (24 crosses and eight parental accessions) before and after cold acclimation. The content of all four sugars (Glc, Fru, Suc, Raf) showed linear correlations with freezing tolerance. Interestingly, however, the sum of all sugars showed a similar correlation. This constitutes novel evidence that the sugars may not have very specific effects, but rather constitute a redundant system of cellular stabilization, or substrates for the synthesis of other cryoprotectants, as hypothesized earlier (Klotke *et al.* 2004). The observed lack of specificity of sugars is in accord with a report that *Arabidopsis* plants that are not able to synthesize Raf, because of a knock-out (k.o.) mutation in the raffinose synthase gene show the same freezing tolerance and cold acclimation behaviour as the wild-type Col plants (Zuther *et al.* 2004), indicating that Raf does not play an indispensable role in *Arabidopsis* freezing tolerance and cold acclimation. Unfortunately, similar studies are not possible with the other three sugars, as they play crucial roles in central metabolism. Clearly, correlations represent no proof for any causality. However, it has been shown earlier that accessions that acclimate poorly nevertheless accumulate large amounts of starch over a 14 d acclimation period (Hannah *et al.* 2006; Guy *et al.* 2008), indicating that it is not the lack of available fixed carbon that limits freezing tolerance and metabolite accumulation. The data presented here on a much wider range of genotypes provide further evidence for a functional role of sugars (if not a particular sugar) in plant freezing tolerance.

A similar picture as seen for freezing tolerance emerged also from our analysis of the involvement of sugars in the heterotic effects observed in freezing tolerance. All four sugars showed MPD and BPD of their contents, especially after cold acclimation, and these were clearly correlated with heterosis in freezing tolerance. The sum of the deviations from mid-parent or best-parent values in the content of the four sugars was correlated to a similar degree with heterosis in freezing tolerance, again indicating redundancy of the different sugars.

Interestingly, the amino acid proline, which is also generally considered a compatible solute (Yancey *et al.* 1982; Somero 1992), presents a different picture. While we could observe a correlation between leaf proline content and freezing tolerance, the deviations from mid-parent and best-parent values were weak, and there were no correlations with heterotic effects in freezing tolerance. This finding clearly shows that the correlations observed for the sugars are not just unspecific effects on all solutes, but that some solutes contribute significantly to the observed heterosis in freezing tolerance, while others do not. This is important evidence to suggest that metabolites, or specific combinations of metabolites, might be identified, which contribute significantly to the observed heterosis in freezing tolerance. A comprehensive metabolite profiling is currently underway to establish the role of the metabolome in the heterosis of *Arabidopsis* freezing tolerance. The data presented here strongly suggest that not all metabolites that accumulate during cold acclimation play a role in the heterosis in freezing tolerance, and therefore, this is a unique experimental system to distinguish the functional roles of different metabolites or groups of metabolites.

The flavonoid biosynthesis pathway in *Arabidopsis* is regulated by the MYB family transcription factors PAP1 and PAP2, which control the regulon of genes encoding the flavonoid biosynthesis pathway enzymes (Tohge *et al.* 2005). The *PAP1* gene is up-regulated by Suc (Teng *et al.* 2005), while *PAP2* is strongly cold induced at the transcript level (Hannah *et al.* 2005, 2006) indicating that although Suc is accumulated in cold-acclimated plants, the induction of flavonoid biosynthesis during cold acclimation follows a separate signalling pathway. The expression of *PAP2* is strongly correlated with the acclimated freezing tolerance of the *Arabidopsis* accessions used in the present study (Hannah *et al.* 2006). However, whether the observed differences in gene expression are reflected in the flavonoid content of leaves, and whether this is also correlated with leaf freezing tolerance, have previously not been investigated. Comprehensive profiling of the flavonoid composition of five accessions and eight F_1_ hybrids, both before and after cold acclimation, showed here for the first time that there is considerable natural variation in *Arabidopsis* flavonol composition. In addition, cold acclimation has a major influence on the amount and composition of flavonols, in agreement with the gene expression data.

The content of all major flavonols was linearly correlated with leaf freezing tolerance. These correlations were in general stronger than those between sugars and freezing tolerance (*r* = 0.83–0.90 versus *r* = 0.66–0.70), indicating that flavonols may indeed play a functional role in plant freezing tolerance. A possible caveat in this comparison is the difference in sample size, because we investigated fewer genotypes for flavonoid than for sugar content. However, when we performed the correlation analyses for the sugar content data with only those genotypes that were also investigated for flavonoid content, we found almost unchanged *r* values, but because of the smaller sample size, higher *P* values (0.0293–0.0824; data not shown). This strongly supports our conclusions and emphasizes the high significance of the correlations observed for the flavonols. This is the first evidence that flavonols may play a functional role in plant cold acclimation and freezing tolerance.

In addition, all major flavonols showed significant deviations from mid-parent and best-parent values in several crosses, especially after cold acclimation. The degree of correlation between MPD and BPD in flavonol content and heterosis in freezing tolerance was much more variable between the different flavonols than between different sugars, indicating that some of the flavonols may play more specific roles in freezing tolerance than the sugars. Especially striking is the fact that both MPD and BPD in the content of the flavonol in peak 8 are much more strongly correlated (higher *r* and lower *P* values) with heterosis in freezing tolerance than the respective values for the content of the flavonol in peak 6, although both peaks contain kaempferol glycosides. The functional basis of this difference is presently unclear, but our results strongly indicate this to be an interesting area of further research.

Several stress-related functions in plants have been proposed for flavonoids in the literature, for example, protection against UV-B radiation and antioxidant activity (see Rice-Evans, Miller & Paganga 1997; Harborne & Williams 2000; Winkel-Shirley 2002 for comprehensive reviews). However, UV-B-absorbing flavonoids are located in the upper epidermal cell layer and in the cuticular wax layer, and would not contribute to the freezing tolerance of other cells in a leaf. In addition, our freezing experiments are performed in the dark, and cold acclimation is performed in climate chambers with artificial lighting that does not produce significant amounts of UV-B radiation. Therefore, protection against UV-B radiation can be excluded as a mechanism contributing to freezing tolerance and cold acclimation in our experiments. However, cold-induced flavonoids have been shown to be effective UV-B screens in a number of different species (Bilger, Rolland & Nybakken 2007), indicating that under natural conditions, this may be an important part of the adaptation processes of cold acclimation.

The scavenging of reactive oxygen species (ROS) during cold acclimation and during freezing may be another protective mechanism afforded by flavonoids, and it has been shown that flavonols such as quercetin are especially potent antioxidants (Rice-Evans *et al.* 1997). Further experiments will be necessary to evaluate the contribution of ROS to freezing damage under our experimental conditions and possible scavenging effects of different flavonols, for example, in the chloroplasts (Agati *et al.* 2007).

In addition to ROS scavenging, flavonols may also have direct effects on the stability of cellular membranes. It has been shown that flavonols can partition into the lipid phase of membranes (Scheidt *et al.* 2004). Under freezing conditions, when a large part of the water is removed from the cells to intercellular ice crystals, it can be expected that amphiphiles such as flavonoids will partition even more strongly into the hydrophobic phase of membranes (Hoekstra & Golovina 2002). While this can be disruptive in some cases (Ollila *et al.* 2002; Tamba *et al.* 2007), it has also been shown for a glucosylated phenol (arbutin; 4-hydroxyphenol-*β*-d-glucopyranoside) that it is able to specifically stabilize membranes that contain non-bilayer lipids (Hincha, Oliver & Crowe 1999, Oliver *et al.* 2001). Similar mechanisms could be envisaged also for flavonoids, but this hypothesis needs to be experimentally tested in the future.

## Supplementary Material

Table S1 Statistical significance of deviations from mid-parent or best-parent values (MPD, mid-parent deviation; BPD, best-parent deviation) in the sugar content of crosses between *Arabidopsis* accessions, indicated as *P* values from *t*-tests. Plants were harvested after growth under non-acclimating conditions (NA), or after an additional 14 d of cold acclimation at 4 °C (ACC). Values in bold indicate *P* values <0.05. n.a., not applicable (significant negative BPD); n.d., not determined.

Table S2 Statistical significance of deviations from mid-parent or best-parent values (MPD, mid-parent deviation; BPD, best-parent deviation) in the flavonol content of crosses between *Arabidopsis* accessions, indicated as *P* values from *t*-tests. Plants were harvested after growth under non-acclimating conditions (NA), or after an additional 14 d of cold acclimation at 4 °C (ACC). The peak numbering refers to the high-performance liquid chromatography (HPLC) elution profile shown in Fig. 9. Further information about the analysed compounds can be found in Table 1. Values in bold indicate *P* values <0.05. n.a., not applicable [significant negative best-parent deviation (BPD)]. This material is available as part of the online article from http://www.blackwell-synergy.com/doi/abs/10.1111/j.1365-3040.2008.01800.x (This link will take you to the article abstract) Please note: Blackwell Publishing is not responsible for the content or functionality of any supplementary materials supplied by the authors. Any queries (other than missing material) should be directed to the corresponding author for the article.
